# Parasitic wasps related to *Prays oleae* (Bernard, 1788) (Lepidoptera, Praydidae) in olive orchards in Greece

**DOI:** 10.3897/zookeys.773.25402

**Published:** 2018-07-09

**Authors:** Eleftherios Alissandrakis, Panagiota Psirofonia, Nickolas G. Kavallieratos, Saša S. Stanković, Vladimir Žikić

**Affiliations:** 1 Laboratory of Entomology and Pesticide Science, Department of Agriculture, Technological Educational Institute of Crete, P.O. Box 1939, 71004, Heraklion, Crete, Greece; 2 Laboratory of Agricultural Zoology and Entomology, Department of Crop Science, Agricultural University of Athens, 75 Iera Odos str., 11855, Athens, Attica, Greece; 3 Department of Biology and Ecology, Faculty of Sciences and Mathematics, University of Niš, Višegradska 33, 18000, Niš, Serbia

**Keywords:** Diptera, Greece, Hymenoptera, parasitoids, *Prays oleae*

## Abstract

The olive moth, *Prays oleae* (Bernard, 1788) (Lepidoptera: Praydidae) is categorised among the most devastating insect pests of olives, whose anthophagous and carpophagous generations can cause yield loss up to 581 and 846 kg of fruit per ha, respectively. In this study, results of the captured parasitoids in olive tree (*Olea europaea* Linnaeus, 1753) orchards, or infested olive plant material in Crete, Greece, is presented. Five of the six identified species captured in trap devices are related to *P. oleae*, i.e., *Chelonus elaeaphilus* Silvestri, 1908, *Chelonus pellucens* (Nees, 1816), *Apanteles xanthostigma* (Haliday, 1834), *Diadegma armillatum* (Gravenhorst, 1829), and *Exochus lentipes* Gravenhorst, 1829. The species *Eupelmus urozonus* Dalman, 1820 and *Pnigalio mediterraneus* Ferrière & Delucchi, 1957 were reared from infested *P. oleae* leaves. *Chelonus pellucens* is reported for the first time from Greece. According to the international literature, 59 hymenopterous and dipterous parasitoid species are associated with *P. oleae* in Europe.

## Introduction

Olive trees growing has been traditionally localised in the Mediterranean Basin for thousands of years, where almost 97.9% of the cultivated areas are located (Rallo et al. 2018). The list of potentially harmful organisms includes more than 255 species and the losses due to insect pests alone are estimated to be approximately 15% of production (Haniotakis 2003). Among them, the most common species are the olive fruit fly, *Bactrocera oleae* (Rossi, 1790) (Diptera: Tephritidae), the olive moth, *Prays oleae* (Bernard, 1788) (Lepidoptera: Praydidae), and the Mediterranean black scale, *Saissetia oleae* (Olivier, 1791) (Hemiptera: Coccidae) (Haniotakis 2003).

*Prays oleae* is one of the main pests infesting olives of commercial production, since larvae of the first, second, and third generations attack flowers, fruits, and leaves, respectively (Kavallieratos et al. 2005; Nave et al. 2017). The anthophagous generation can cause yield losses up to 581 kg of fruit per ha and the corresponding carpophagous up to 846 kg per ha, an issue that justifies the imposed control measures (Bento et al. 2001). In recent years, high socioeconomic pressures have forced olive growers to develop alternative control strategies in an effort to mitigate the undesirable side effects of pesticides on trophic chains and biological balances (Nave et al. 2017). In this sense, not only the economic losses due to the pest should be evaluated, but also the possible secondary effects that such control measures can have on beneficial fauna (Ramos et al. 1998).

Previous research has revealed a wide parasitoid spectrum that is related to *P. oleae*, resulting to biological control efforts against this pest. The first parasitoid used in biocontrol program was *Trichogramma embryophagum* (Hartig, 1838) (Hymenoptera: Trichogrammatidae) in former Yugoslavia (Brnetić 1988). In Spain, three species have been released against *P. oleae* with various levels of success; i.e., *Chelonus elaeaphilus* Silvestri, 1908 (Hymenoptera: Braconidae), the specialised Ageniaspis fuscicollis (Dalman, 1820) var. praysincola Silvestri, 1907 (Hymenoptera: Encyrtidae) and *T. embryophagum* (Civantos and Caballero 1993). *Trichogramma cacaeciae* Marchal, 1927 (Hymenoptera: Trichogrammatidae) has been utilised in Portugal (Bento et al. 1998) and *Trichogramma evanescens* Westwood, 1833 in Egypt (Agamy 2010).

Although there are previous records concerning the occurrence of *P. oleae* parasitoids in Greece, there are no data available from the island of Crete, the most important olive production area with almost 200,000 ha cultivated with olive trees (i.e., nearly 25% of the total island area is covered with olive plantations; Hellenic Statistical Authority 2014). Given that the knowledge of the beneficial entomofauna of the olive crop is clearly linked with the biological control of pests infesting this crop and that indigenous strains of parasitoids occurring in olive groves can be more effective against certain olive pests than the commercially available parasitoids (Herz and Hassan 2006), the objective of this study was to further investigate the parasitoid complex that is associated with *P. oleae* in the overlooked area of Crete by using trap devices and collecting plant material.

## Materials and methods

All parasitoids were collected in olive orchards from the island of Crete, Greece from June to October 2017. A part of the material was captured in five glass McPhail trap devices, installed from June to October in an olive orchard at Messara (Crete) that covers an area of approx. 0.5 ha baited with 200 ml aqueous solution of 2% hydrolysed protein (Entomela 75 SL, 25% w/w urea; BASF Hellas, Amaroussion, Greece). Each trap device was placed with its lower part at a height of 2 m from the ground. The distances among trap devices were approx. 100 m. The solution was replaced every week. Additional specimens were reared from *P. oleae* infested plant material (O. europaea var. koroneiki). Infested leaves by *P. oleae* larvae were collected from olive trees, separately transferred into plastic vials covered with mesh, and transferred to the laboratory. Vials were maintained at 25 °C and 60% relative humidity and inspected daily for emergence of parasitoids. All parasitoid individuals, either from trap devices or plant material, were preserved in 96% alcohol. Specimens were dissected and slide mounted in Berlese medium. The identification of the captured and reared specimens was conducted under a Nikon SM2 745T binocular stereomicroscope (Nikon CEE GmbH, Wien, Austria) or an Olympus SZX9 (Olympus Corporation, Tokyo, Japan) using appropriate keys (Tobias et al. 1986; Askew and Nieves Aldrey 2000; Tolkanitz 2007; Broad 2011). Part of the specimens was deposited in the insect collection of the Laboratory of Agricultural Zoology of Entomology, Agricultural University of Athens, Greece, and a part was deposited in the insect collection of the Faculty of Sciences and Mathematics, Department of Biology and Ecology, University of Niš, Serbia.

Additional to field research, we critically reviewed all recorded parasitoids of *P. oleae* in Greece and Europe indicating the pest’s stage they attack. The synonymy among taxa was checked and adopted according to online databases (van Achterberg 2013; Fernandez Triana and Ward 2015; Noyes 2017; Tschorsnig 2017), and the database provided by Yu et al. (2012).

## Results

In total, five out of six species captured in McPhail trap devices are related to *P. oleae*, i.e., *C. elaeaphilus*, *Chelonus pellucens* (Nees, 1816) (Hymenoptera: Braconidae), *Apanteles xanthostigma* (Haliday, 1834) (Hymenoptera: Braconidae), *Diadegma armillatum* (Gravenhorst, 1829) (Hymenoptera: Ichneumonidae), and *Exochus lentipes* Gravenhorst, 1829 (Hymenoptera: Ichneumonidae), while two species were reared from *P. oleae* infested olive leaves.

The exhaustive investigation of the international literature revealed 59 hymenopterous and dipterous parasitoid species that attack *P. oleae* in Europe; 14 Braconidae, 2 Chalcididae, 1 Encyrtidae, 20 Eulophidae, 1 Eupelmidae, 7 Icheumonidae, 1 Platygastridae, 3 Pteromalidae, 2 Tachinidae, and 8 Trichogrammatidae (Table 1). Thirty-one out of these 59 parasitoid species have been recorded in Greece: 9 Braconidae, 1 Chalcididae, 1 Encyrtidae, 13 Eulophidae, 1 Eupelmidae, 3 Icheumonidae, 1 Platygastridae, and 2 Trichogrammatidae. All Braconidae, Chalcididae, Encyrtidae, Eupelmidae, Platygastridae, and Tachinidae which are parasitoids of *P. oleae* attack only larvae. All eulophids parasitise larvae of *P. oleae* while some of them attack both larvae and pupae. Three icheumonids parasitise larvae exclusively and four both larvae and pupae. All pteromalids are pupal parasitoids whilst all trichogrammatids are egg parasitoids.

**Table 1.** Parasitoids of *Prays oleae* recorded in Europe and their presence in Greece: (+) recorded, (-) not recorded.

Family	Species	Source of host record	Host stage attacked	Recorded or not in Greece
Braconidae	*Aleiodes circumscriptus* (Nees, 1834)	Beyarslan (2015)	larva	+
	*Aleiodes gastritor* (Thunberg, 1822)	Halperin (1986)	larva	+
	*Apanteles xanthostigma* (Haliday, 1834)	Nave et al. (2016)	larva	+
	*Bracon hebetor* Say, 1836	Falco et al. (1993)	larva	+
	*Bracon laetus* (Wesmael, 1838)	Aubert (1966)	larva	+
	*Bracon crassicornis* Thomson, 1892	Silvestri (1908)	larva	+
	Chelonus (Microchelonus) elaeaphilus Silvestri, 1908	Nave et al. (2016)	larva	+
	Chelonus (Microchelonus) silvestrii (Papp, 1999)	Papp (1999)	larva	-
	*Chelonus* (*Parachelonus) pellucens* (Nees, 1816)	Texeira et al. (2000)	larva	-
	*Clinocentrus testaceus* (Kriechbaumer, 1894)	Texeira et al. (2000)	larva	-
	*Dolichogenidea dilecta* (Haliday, 1834)	Telenga (1955)	larva	-
	*Dolichogenidea ultor* (Reinhard, 1880)	Arambourg (1969)	larva	-
	*Meteorus rubens* (Nees, 1811)	Texeira et al. (2000)	larva	+
	*Phanerotoma dentata* (Panzer, 1805)	Texeira et al. (2000)	larva	+
Chalcididae	*Hockeria bifasciata* Walker, 1834	Madl (2008)	larva	-
	*Hockeria unicolor* Walker, 1834	Stavraki (1977)	larva	+
Encyrtidae	*Ageniaspis fuscicollis* (Dalman, 1820) var. praysincola Silvestri, 1907	Nave et al. (2016)	larva	+
Eulophidae	*Asecodes erxias* (Walker, 1848)	Silvestri (1908)	larva	+
	*Baryscapus nigroviolaceus* (Nees, 1834)	Noyes (2017)	larva	-
	*Chrysocharis gemma* (Walker, 1839)	Noyes (2017)	larva	+
	*Chrysocharis nephereus* (Walker, 1839)	Noyes (2017)	larva	+
	*Cirrospilus elongatus* Boucek, 1959	Noyes (2017)	larva	-
	*Dicladocerus westwoodii* Westwood, 1832	Ramos and Panis (1975)	larva	+
	*Elasmus arcuatus* Ferrière, 1947	Ferrière (1947)	larva	-
	*Elasmus flabellatus* (Fonscolombe, 1832)	Nave et al. (2016)	larva	+
	*Elasmus masii* Ferrière, 1929	Anonymous (2006)	larva	-
	*Elasmus nudus* (Nees, 1834)	Ramos and Panis (1975)	larva	-
	*Elasmus steffani* Viggiani, 1967	Redolfi and Campos (2010)	larva	+
	*Elasmus westwoodi* Giraud, 1856	Noyes (2017)	larva	+
	*Euderus albitarsis* (Zetterstedt, 1838)	Nave et al. (2016)	larva	-
	*Hemiptarsenus unguicellus* (Zetterstedt, 1838)	Noyes (2017)	larva	-
	*Pediobius bruchicida* (Rondani, 1872)	Bouček (1974)	larva/ pupa	+
	*Pnigalio agraules* (Walker, 1839)	Nave et al. (2016)	larva/ pupa	+
	*Pnigalio epilobii* Bouchek, 1966	Stavraki (1970)	larva/ pupa	+
	*Pnigalio longulus* (Zetterstedt, 1838)	Stavraki (1970)	larva/ pupa	+
	*Pnigalio mediterraneus* Ferrière & Delucchi, 1957	Stavraki (1970)	larva/ pupa	+
	*Pnigalio pectinicornis* (Linnaeus, 1758)	Ramos and Panis (1975)	larva/ pupa	+
Eupelmidae	*Eupelmus urozonus* Dalman, 1820	Noyes (2017)	larva	+
Ichneumonidae	*Diadegma armillatum* (Gravenhorst, 1829)	Bento et al. (1998)	larva/ pupa	+
	*Diadegma semiclausum* (Hellén, 1949)	Torres (2010)	larva/ pupa	+
	*Exochus lentipes* Gravenhorst, 1829	Texeira et al. (2000)	larva	-
Ichneumonidae	*Himertosoma superbum* Schmiedeknecht, 1900	Vidal (1997)	larva/ pupa	-
	*Itoplectis alternans* (Gravenhorst, 1829)	Silvestri (1908)	larva/ pupa	-
	*Lissonota superbator* Aubert, 1967	Aubert (1969)	larva	+
	*Scambus elegans* (Woldstedt, 1877)	Nave et al. (2017)	larva	-
Platygastridae	*Platygaster apicalis* Thomson, 1859	Stavraki (1970)	larva	+
Pteromalidae	*Mesopolobus mediterraneus* (Mayr, 1903)	Bozbuğa and Elekçioğlu (2008)	pupa	-
	*Pteromalus chrysos* Walker, 1836	Noyes (2017)	pupa	-
	*Pteromalus semotus* Walker, 1834	Noyes (2017)	pupa	-
Tachinidae	*Phytomyptera nigrina* (Meigen, 1824)	Kara and Tschorsnig (2003)	larva	-
	*Phytomyptera vaccinii* Sintenis, 1897	Tschorsnig (2017)	larva	-
Trichogrammatidae	*Trichogramma bourarachae* Pintureau & Babault, 1988	Polaszek (2009)	egg	-
	*Trichogramma brassicae* Bezdenko, 1968	Polaszek (2009)	egg	-
	*Trichogramma cordubensis* Vargas & Cabello, 1985	Jardak (1980)	egg	-
	*Trichogramma dendrolimi* Matsumura, 1926	Polaszek (2009)	egg	-
	*Trichogramma euproctidis* (Girault, 1911)	Pereira et al. (2004)	egg	+
	*Trichogramma minutum* Riley, 1871	Stavraki (1985)	egg	-
	*Trichogramma oleae* Voegele & Pointel, 1979	Polaszek (2009)	egg	+
	*Trichogramma pretiosum* Riley, 1879	Polaszek (2009)	egg	-

**Family Braconidae**

***Apanteles xanthostigma* (Haliday, 1834)**

**Material examined**: 11 ♀, Messara (Crete) (35°2'20"N, 24°50'54"E), 16–23.06.2017, captured in McPhail trap device.

**Chelonus (Microchelonus) elaeaphilus (Silvestri, 1907)**

**Material examined**: 4 ♀, 4 ♂, Messara (Crete) (35°2'20"N, 24°50'54"E), 09–16.06.2017, captured in McPhail trap device.

**Chelonus (Parachelonus) pellucens (Nees, 1816)**

**Material examined**: 6 ♀, Messara (Crete) (35°2'20"N, 24°50'54"E), 09–16.06.2017, captured in McPhail trap device.

***Glyptapanteles vitripennis* (Curtis, 1830)**

**Material examined**: 2 ♀, 7 ♂, Messara (Crete) (35°2'20"N, 24°50'54"E), 23–30.06.2017, captured in McPhail trap device.

**Family Eulophidae**

***Pnigalio mediterraneus* Ferrière & Delucchi, 1957**

**Material examined**: 12 ♂, Heraklion, Voutes, (Crete) (35°15'54"N, 25°03'26"E), 15.03.2017 (date of host collection). Host: *Prays oleae* on Olea europaea var. koroneiki.

**Family Eupelmidae**

***Eupelmus urozonus* Dalman, 1820**

**Material examined**: 8 ♀, 12 ♂, Heraklion, Voutes, (Crete) (35°15'54"N, 25°03'26"E), 15.03.2017 (date of host collection). Host: *Prays oleae* on Olea europaea var. koroneiki.

**Family Ichneumonidae**

***Diadegma armillatum* (Gravenhorst, 1829)**

**Material examined**: 3 ♀, 5 ♂, Messara (Crete) (35°2'20"N, 24°50'54"E), 16–23.08.2017, captured in McPhail trap device.

***Exochus lentipes* Gravenhorst, 1829**

**Material examined**: 6 ♀, 8 ♂, Messara (Crete) (35°2'20"N, 24°50'54"E), 16–23.09.2017, captured in McPhail trap device.

## Discussion

Microgastrinae is one of the largest subfamilies of Braconidae with about 2,000 described species worldwide (Pérez Rodríguez et al. 2013). Very recently, the hymenopteran parasitoid complex of *P. oleae* was studied in Portugal where, among the 22 recorded parasitoid taxa, *A. xanthostigma* was the major natural enemy (Nave et al. 2017). Furthermore, in Egypt *A. xanthostigma* was found to parasitise the larval stage of *P. oleae* at a rate of more than 50% (Herz et al. 2005). Apart from *P. oleae*, this parasitoid species parasitises a high number of microlepidopterous species, mainly Tortricidae, Gracillariidae, and Yponomeutidae, particularly the genera *Paraswammerdamia* Friese, 1960 and *Swammerdamia* Hübner, 1825 (Yu et al. 2012). *Glyptapanteles* Ashmead, 1904 is a genus with about 200 species in Palaearctic and Nearctic regions and, like all Microgastrinae, are koinobiont endoparasitoids of lepidopteran larvae (Penteado Dias et al. 2011). *Glyptapanteles vitripennis* was first reported in southern Greece in 1978 (Papp 2007) without further records since then. This parasitoid species was the second most abundant recovered from Malaise traps placed in the Artikutza forest of Pyrenees (Spain) (Pérez Rodríguez et al. 2013) while it is also known that it attacks *Yponomeuta malinellus* (Zeller, 1838) (Lepidoptera: Yponomeutidae) (Velcheva et al. 2012). Given that this species parasitises numerous other lepidopterous species belonging to Geometridae, Noctuidae, Plutellidae, and Tortricidae (Nixon 1973), it could be a good candidate for biological control purposes. Whether *G. vitripennis* parasitises *P. oleae*, it remains to be confirmed with additional field efforts.

The subfamily Cheloninae is formed by more than 1,300 species belonging to 15 genera, thus constituting a quite large part of Braconidae (Kittel and Austin 2014). They oviposit into eggs and larvae of various lepidopterous species, a fact that makes them valuable potential biocontrol agents (Inayatullah and Naeem 2004; Walker and Huddleston 1987; Edmardash et al. 2011). The subgenus Microchelonus Szepligeti, 1908 is even considered as a valid genus, following the standpoints of Papp (2014a, b). The genus *Chelonus* Panzer, 1806 counts 601 species in the Holarctic region (Papp 2014c) with *M. elaeaphilus* being known in the Mediterranean region, either as *M. elaeaphilus* or *C. elaeaphilus* (Papp 2012; Nave et al. 2017). This species has been introduced and established in Greece from France (Yamvrias 1998). On the other hand, *C. pellucens* has a wider European distribution than *M. elaeaphilus* (van Achterberg 2013). *Chelonus pellucens* is reported for the first time from Greece and although *C. elaeaphilus* parasitises *P. oleae* (Bento et al. 1998), there are no relevant records for *C. pellucens*, an issue that merits further investigation.

Although Eupelmidae is a relatively small family with approximately 1000 species, the genus *Eupelmus* Dalman, 1820 is a large taxon containing more than 300 species (Gibson and Fusu 2016) whilst Eulophidae is one of the largest families within chalcidoid wasps, with almost 5,000 species (Aguiar et al. 2013). The genus *Pnigalio* Schrank, 1802 is comprised by 61 valid species (Li et al. 2017). Several hosts of *Pnigalio mediterraneus* Ferrière & Delucchi, 1957 (Hymenoptera: Eulophidae) are major pests of plants of ornamental and agricultural importance belonging to different orders, such as *B. oleae*, *Phyllocnistis citrella* Stainton, 1856 (Lepidoptera: Gracillariidae), and *Cameraria ohridella* Deschka & Dimić, 1986 (Lepidoptera: Gracillariidae) (Gebiola et al. 2009). Both *Eupelmus urozonus* Dalman, 1820 (Hymenoptera: Eupelmidae) and *P. mediterraneus* were found in the Greek island of Corfu as primary parasitoids of *B. oleae* (Kapatos and Fletcher 1986). Based on our results, these species are also parasitoids of *P. oleae* that occur in Greece since they were recorded from infested olive leaves.

The genus *Diadegma* Förster, 1869 constitutes a large group of Ichneumonid wasps with more than 200 known species worldwide (Wagener et al. 2006). *Diadegma armillatum* is a known parasitoid of various lepidopterous species (Velcheva et al. 2012; Fernandez Triana et al. 2014) that has been recently recorded attacking *P. oleae* larvae (Nave et al. 2017). The genus *Exochus* Gravenhorst, 1829 is the largest group of Metopiinae including the widely distributed in Europe, *E. lentipes* that attacks various Tortricidae and Gelechiidae larvae (Yu et al. 2012).

Our original findings on associated parasitoids of *P. oleae* and the compiled information revealed could trigger further studies that deal with the management of this noxious insect species in the target area from a biological control point of view. The identified parasitoid spectrum was broad, despite the short interval of obtaining the data, indicating a potential positive impact of natural enemies to *P. oleae*, an issue however that merits further field efforts. Last but not least, given that *C. pellucens* is identified as a new member of the entomofauna of Greece during the present first attempt to record the beneficial parasitoids in olive orchards in Crete, we may expect that additional parasitoid species may occur in this agroecosystem.

## References

[B1] Agamy E (2010) Field evaluation of the egg parasitoid, *Trichogramma evanescens* West. against the olive moth *Prays oleae* (Bern.) in Egypt. Journal of Pest Science 83: 53–58. 10.1007/s10340-009-0273-x

[B2] Aguiar AP Deans AR Engel MS Forshage M Huber JT Jennings JT Johnson NF Lelej AS Longino JT Lohrmann V Mikó I Ohl M Rasmussen C Taeger A Yu DSK (2013) Order Hymenoptera. Zootaxa 3703: 51–62. 10.11646/zootaxa.3703.1.12

[B3] Anonymous (2006) *Prays* del olivo (*Prays oleae*). Boletín Phytosanitario 6: 1–16.

[B4] Arambourg Y (1969) Inventaire de la biocœnose parasitaire de *Prays oleae* dans le bassin méditerranéen. Entomophaga 14: 185–194. 10.1007/BF02371159

[B5] Askew RR Nieves Aldrey JL (2000) The genus *Eupelmus* Dalman, 1820 (Hymenoptera: Chalcidoidea: Eupelmidae) in peninsular Spain and the Canary Islands, with taxonomic notes and descriptions of new species. Graellsia 56: 49–61. 10.3989/graellsia.2000.v56.i0.309

[B6] Aubert JF (1966) Liste d’identification No. 6 (présentée par le service d’identification des Entomophages). Entomophaga 11: 115–134. 10.1007/BF02371463

[B7] Aubert JF (1969) Les Ichneumonides ouest-paléarctiques et leurs hôtes, Volume I Pimplinae, Xoridinae, Acaenitinae. Éditions Quatrefeuilles, Paris, 302 pp.

[B8] Bento A Torres LM Lopes J (2001) Avaliação de prejuízos causados pela traça da oliveira, *Prays oleae* (Bern.) em Trás-os-Montes. Revista de Ciências Agrárias 24: 89–96.

[B9] Bento A Ilideo J Campos M Torres L (1998) Parasitismo associado à traça da oliveira *Prays oleae* Bern. em Trás-os-Montes (Nordeste de Portugal). Boletín de Sanidad Vegetal Plagas 24: 949–954.

[B10] Beyarslan A (2015) Taxonomic survey on the Rogadinae Förster, 1862 (Hymenoptera: Braconidae) in the northeastern Anatolian region, Turkey. Turkish Journal of Zoology 39: 811–819. https://10.3906/zoo-1407-35

[B11] Bouček Z (1974) On the Chalcidoidea (Hymenoptera) described by C. Rondani. Redia 55: 241–285.

[B12] Bozbuğa R Elekçioğlu Z (2008) Pests and natural enemies determined in olive orchards in Turkey. Türk Bilimsel Derlemeler Dergisi 1: 87–97.

[B13] Brnetić D (1988) Biological control of the olive pests in the Yugoslav littoral. Olea 19: 92–93.

[B14] Broad G (2011) Identification key to the subfamilies of Ichneumonidae (Hymenoptera). Natural History Museum, London, 40 pp.

[B15] Civantos M Caballero JM (1993) Integrated pest management in olive in the Mediterranean area. EPPO Bulletin 23: 367–375. 10.1111/j.1365-2338.1993.tb01338.x

[B16] Edmardash YAE Abdel Dayem MS Gadallah NS (2011) The subfamily Cheloninae (Hymenoptera: Braconidae) from Egypt, with the description of two new species. ZooKeys 115: 85–102. 10.3897/zookeys.115.1186 PMC3187663 21977002

[B17] Falcó JV Monero J Jiménez R (1993) Datos sobre Ciclostominos ibéricos. I. Braconinae (Hymenoptera: Braconidae). Boletin de la Associacion Espanola de Entomologia 17: 71–90.

[B18] Fernandez Triana J Ward D (2015) Microgastrinae wasps of the world. http://microgastrinae.myspecies.info/ [accessed on 3 January 2018]

[B19] Fernandez Triana J Shaw MR Cardinal S Mason PG (2014) Contributions to the study of the Holarctic fauna of Microgastrinae (Hymenoptera: Braconidae). I. Introduction and first results of transatlantic comparisons. Journal of Hymenoptera Research 37: 61–76. 10.3897/jhr.37.7186

[B20] Ferrière C (1947) Les espèces européennes du genre *Elasmus* Westw. (Hym. Chalc.). Mitteilungen der Schweizerischen Entomologischen Gesellschaft 20: 565–580.

[B21] Gebiola M Bernardo U Monti MM Navone P Viggiani G (2009) *Pnigalio agraules* (Walker) and *Pnigalio mediterraneus* Ferrière and Delucchi (Hymenoptera: Eulophidae): two closely related valid species. Journal of Natural History 43: 2465–2480. 10.1080/00222930903105088

[B22] Gibson GAP Fusu L (2016) Revision of the Palaearctic species of Eupelmus (Eupelmus) Dalman (Hymenoptera: Chalcidoidea: Eupelmidae). Zootaxa 4081: 1–331. 10.11646/zootaxa.4081.1.1 27394215

[B23] Halperin J (1986) Braconidae (Hymenoptera) associated with forest and ornamental trees and shrubs in Israel. Phytoparasitica 14: 119–135. 10.1007/BF02980898

[B24] Haniotakis GE (2003) Olive pest control: present status and prospects. IOBC Bulletin 28: 1–9.

[B25] Hellenic Statistical Authority (2014) Hellenic Statistical Authority. http://www.statistics.gr/en/home/ [accessed on 26 March 2018]

[B26] Herz A Hassan SA (2006) Are indigenous strains of *Trichogramma* sp. (Hymenoptera: Trichogrammatidae) better candidates for biological control of lepidopterous pests of the olive tree? Biocontrol Science and Technology 16: 841–857. 10.1080/09583150600827751

[B27] Herz A Hassan SA Hegazi E Khafagi WE Nasr FN Youssef AA Agamy E Jardak T Ksantini M Mazomenos BE Konstantopoulou MA Torres L Gonçalves F Bento A Pereira JA (2005) Towards sustainable control of lepidopterous pests in olive cultivation. Gesunde Pflanzen 58: 117–128. 10.1007/s10343-005-0076-9

[B28] Inayatullah M Naeem M (2004) An identification key to genera of Cheloninae (Braconidae: Hymenoptera) in the NWFP with new distributional records and taxonomic notes. Sarhad Journal of Agriculture 20: 143–147.

[B29] Jardak T (1980) Etudes bioécologiques de *Prays oleae* BERN (Lepidoptera: Hyponomeutidae) et de ses parasites oophages du genre *Trichogramma* (Hymenoptera: Trichogrammatidae): essais d’utilisation en lutte biologique. Thèse de 3éme cycle, Université d’Aix Marseille, Marseille, 42 pp.

[B30] Kapatos ET Fletcher BS (1986) Mortality factors and life-budgets for immature stages of the olive fly, *Dacus oleae* (Gmel.) (Diptera: Tephritidae), in Corfu. Journal of Applied Entomology 102: 326–342. 10.1111/j.1439-0418.1986.tb00931.x

[B31] Kara K Tschorsnig HP (2003) Host catalogue for the Turkish Tachinidae (Diptera). Journal of Applied Entomology 127: 465–476. 10.1046/j.0931-2048.2003.00786.x

[B32] Kavallieratos NG Athanassiou CG Balotis GN Tatsi GTh Mazomenos BE (2005) Factors affecting male *Prays oleae* (Lepidoptera: Yponomeutidae) captures in pheromone-baited traps in olive orchards. Journal of Economic Entomology 98: 1499–1505. 10.1603/0022-0493-98.5.1499 16334316

[B33] Kittel RN Austin AD (2014) Synopsis of Australian chelonine wasps (Hymenoptera: Braconidae: Cheloninae) with description of two new genera. Austral Entomology 53: 183–202. 10.1111/aen.12070

[B34] Li T Yang ZQ Sun SP Wang R (2017) A new species of *Pnigalio* (Hymenoptera: Eulophidae) parasitizing *Eriocrana semipurpurea alpina* (Lepidoptera: Eriocraniidae) in China, with its biology and a key to Chinese known species. ZooKeys 687: 149–159. 10.3897/zookeys.687.14903 PMC5672578 29114170

[B35] Madl M (2008) Zur Kenntnis der Familie Chalcididae (Hymenoptera: Chalcidoidea) in Österreich. Entomofauna 29: 69–80.

[B36] Nave A Gonçalves F Crespí AL Campos M Torres L (2016) Evaluation of native plant flower characteristics for conservation biological control of *Prays oleae*. Bulletin of Entomological Research 106: 249–257. 10.1017/S0007485315001091 26780918

[B37] Nave A Gonçalves F Teixeira R Amaro Costa C Campos M Torres L (2017) Hymenoptera parasitoid complex of *Prays oleae* (Bernard) (Lepidoptera: Praydidae) in Portugal. Turkish Journal of Zoology 41: 502–512. 10.3906/zoo-1603-50

[B38] Nixon GE (1973) A revision of the north-western European species of the *vitripennis*, *pallipes*, *octonarius*, *triangulator*, *formosus*, *parasitellae*, *matacarpalis* and *circumscriptus*-groups of *Apanteles* Förster (Hymenoptera: Braconidae). Bulletin of Entomological Research 63: 169–228. 10.1017/S0007485300039006

[B39] Noyes JS (2017) Universal Chalcidoidea database. http://www.nhm.ac.uk/our-science/data/chalcidoids/introduction.html/ [accessed on 3 January 2018]

[B40] Papp J (1999) Redescription of F. Silvestri’s two chelonine species (Hymenoptera: Braconidae: Cheloninae). Bollettino del Laboratorio di Entomologia Agraria Filippo Silvestri 55: 15–44.

[B41] Papp J (2007) Braconidae (Hymenoptera) from Greece, 6. Notes fauniques de Gembloux 60: 99–127.

[B42] Papp J (2012) A contribution to the braconid fauna of Israel (Hymenoptera: Braconidae), 3. Israel Journal of Entomology 41–42: 165–219.

[B43] Papp J (2014a) Faunistic contributions to the *Microchelonus* Szépligeti species of the Palaearctic region, with descriptions of two new species (Hymenoptera: Braconidae: Cheloninae). Acta Zoologica Hungaricae 60: 325–358. https:/real.mtak.hu/id/eprint/24326

[B44] Papp J (2014b) First survey of the Neotropical species of *Microchelonus* Szépligeti with descriptions of twenty-five new species (Hymenoptera: Braconidae: Cheloninae). Acta Zoologica Academiae Scientiarum Hungaricae 62: 217–344. 10.17109/AZH.62.3.217.2016

[B45] Papp J (2014c) *Microchelonus deplanus* sp. n. from Canada and checklists of the Nearctic and Palaearctic species of the genus *Microchelonus* Szépligeti, 1908 (Hymenoptera: Braconidae: Cheloninae). Natura Somogyiensis 25: 115–140.

[B46] Penteado Dias AM Fernandes LBR Iemma LGR Dias MM (2011) First occurrence of Protapanteles (Protapanteles) enephes (Nixon, 1965) (Hymenoptera: Braconidae: Microgastrinae) in Brazil and new biological data. Brazilian Journal of Biology 71: 735–738. 10.1590/S1519-69842011000400019 21881798

[B47] Pereira JA Bento A Cabanas JE Torres LM Herz A Hassan SA (2004) Ants as predators of the egg parasitoid *Trichogramma cacoeciae* (Hymenoptera: Trichogrammatidae) applied for biological control of the olive moth, *Prays oleae* (Lepidoptera: Plutellidae) in Portugal. Biocontrol Science and Technology 14: 653–664. 10.1080/09583150410001682386

[B48] Pérez Rodríguez J Oltra Moscardó T Peris Felipo FJ Jiménez Peydró R (2013) Microgastrinae (Hymenoptera: Braconidae) in the forest state of Artikutza (Navarra: Spain): diversity and community structure. Insects 4: 493–505. 10.3390/insects4030493 26462432 PMC4553478

[B49] Polaszek A (2009) Species diversity and host associations of *Trichogramma* in Eurasia. In: Consoli FL Parra JRP Zucchi RA (Eds) Egg parasitoids in agroecosystems with emphasis on Trichogramma. Springer, Dordrecht, 237–266. 10.1007/978-1-4020-9110-0_9

[B50] Rallo L Díez CM Morales Sillero A Miho H Priego Capote F Rallo P (2018) Quality of olives: A focus on agricultural preharvest factors. Scientia Horticulturae 233: 491–509. 10.1016/j.scienta.2017.12.034

[B51] Ramos P Panis A (1975) Les chalcidiens parasites de *Prays oleae* (Lepidoptera: Plutellidae) en Andalousie. Entomophaga 20: 225–227. 10.1007/BF02371946

[B52] Ramos P Campos M Ramos JM (1998) Long-term study on the evaluation of yield and economic losses caused by *Prays oleae* Bern. in the olive crop of Granada (southern Spain). Crop Protection 17: 645–647. 10.1016/S0261-2194(98)00065-9

[B53] Redolfi I Campos M (2010) Developmental and reproductive biology of the ectoparasitoid, *Elasmus steffani*, in a substitute host, *Ephestia kuehniella* Journal of Insect Science 10: 119. 10.1673/031.010.11901 PMC3016709 20874600

[B54] Silvestri F (1908) La tignola dell’olivo (*Prays oleellus* Fabr.). Bollettino del Laboratorio di Zoologia Generale 2: 83–184.

[B55] Stavraki H (1970) Contribution a l'inventaire du complexe parasitaire de quelques insectes nuisibles a l'olivier en Grèce. Entomophaga 15: 225–231. 10.1007/BF02371000

[B56] Stavraki HG (1977) Results obtained from released of the oophagous parasites *Trichogramma* spp. against *Prays oleae* Bern. (Lepidoptera: Hyponomeutidae) over a four year period in Greece. Mededelingen van de Faculteit Landbouwwetenschappen, Rijksuniversiteit Gent 42: 1361–1371.

[B57] Stavraki HG (1985) Use of *Trichogramma* spp. against the carpophagous generation of *Prays oleae* (Bern.) in Greece. In: Cavalloro R, Crovetti A (Eds) Proceedings of the CEC/FAO/IOBC international joint meeting, integrated pest control in olive-groves, Pisa (Italy), 3–6 April 1984, AA Balkema, Rotterdam, 242–246.

[B58] Telenga NA (1955) Braconidae, subfamily Microgasterinae, subfamily Agathinae Fauna USSR, Hymenoptera 5: 311.

[B59] Texeira R Bento A Gonçalves M (2000) Avaliaçao da fauna auxiliar associada ao olival em produçao biológica em Trás-os-Montes. Boletín de Sanidad Vegetal. Plagas 26: 629–636.

[B60] Tobias VI Belokobylskij SA Kotenko AG Medvedev G (1986) Family Braconidae. Keys to the insects of the European part of the USSR, Volume III. Part 5. Nauka Publisher, Leningrad, 500 pp.

[B61] Tolkanitz VI (2007) Ichneumon flies of the genus *Exochus* Gravenhorst (Hymenoptera: Ichneumonidae: Metopiinae) of the fauna of Palaearctic region. Russian Entomological Journal 16: 339–358.

[B62] Torres MR (2010) Parasitoides de plagas identificados en la provincia de Jaén (España). Boletín de la Sociedad Entomológica Aragonesa 46: 597–601.

[B63] Tschorsnig HP (2017) Preliminary host catalogue of Palaearctic Tachinidae (Diptera). http://www.nadsdiptera.org/Tach/WorldTachs/CatPalHosts/Cat_Pal_tach_hosts_Ver1.pdf [accessed on 12 June 2018]

[B64] van Achterberg K (2013) Fauna Europaea: Hymenoptera, Braconidae Fauna Europaea version 2.6.2. http://faunaeur.org/ [accessed on 3 January 2018]

[B65] Velcheva N Atanassov A Karadjova O Hubenov Z (2012) Parasitoid assemblages isolated from externally feeding lepidopterans and codling moth (*Cydia pomonella* L., Tortricidae) in a young apple orchard in West Bulgaria. Bulgarian Journal of Agricultural Science 18: 675–681.

[B66] Vidal S (1997) Determination list of entomophagous insects. No. 13. OILB, Avignon, 53 pp.

[B67] Wagener B Reineke A Löhr B Zebitz CPW (2006) Phylogenetic study of *Diadegma* species (Hymenoptera: Ichneumonidae) inferred from analysis of mitochondrial and nuclear DNA sequences. Biological Control 37: 131–140. 10.1016/j.bopcontrol.2006.01.004

[B68] Walker AK Huddleston T (1987) New Zealand chelonine braconid wasps (Hymenoptera). Journal of Natural History 21: 339–361. 10.1080/00222938700771061

[B69] Yamvrias C (1998) Insect pests of olives. Stamoulis, Athens, 126 pp.

[B70] Yu DS van Achterberg C Horstmann K (2012) Taxapad 2012, Ichneumonoidea 2011. Database on flash-drive. Ottawa, Ontario.

